# Interferon-Gamma Promotes Infection of Astrocytes by *Trypanosoma cruzi*

**DOI:** 10.1371/journal.pone.0118600

**Published:** 2015-02-19

**Authors:** Rafael Rodrigues Silva, Rafael M. Mariante, Andrea Alice Silva, Ana Luiza Barbosa dos Santos, Ester Roffê, Helton Santiago, Ricardo Tostes Gazzinelli, Joseli Lannes-Vieira

**Affiliations:** 1 Laboratório de Biologia das Interações, Instituto Oswaldo Cruz—Fiocruz, Rio de Janeiro, Brasil; 2 Laboratório Multidisciplinar de Apoio à Pesquisa, Departamento de Medicina Clínica, Universidade Federal Fluminense, Rio de Janeiro, Brasil; 3 Departamento de Patologia, Faculdade de Medicina, Universidade Federal Fluminense, Rio de Janeiro, Brasil; 4 Laboratório de Imunologia Celular e Molecular, Centro de Pesquisas René Rachou—Fiocruz, Minas Gerais, Brasil; 5 Departamento de Bioquímica e Imunologia, Instituto de Ciências Biológicas, Universidade Federal de Minas Gerais, Minas Gerais, Brasil; Federal University of São Paulo, BRAZIL

## Abstract

The inflammatory cytokine interferon-gamma (IFNγ) is crucial for immunity against intracellular pathogens such as the protozoan parasite *Trypanosoma cruzi*, the causative agent of Chagas disease (CD). IFNγ is a pleiotropic cytokine which regulates activation of immune and non-immune cells; however, the effect of IFNγ in the central nervous system (CNS) and astrocytes during CD is unknown. Here we show that parasite persists in the CNS of C3H/He mice chronically infected with the Colombian *T. cruzi* strain despite the increased expression of IFNγ mRNA. Furthermore, most of the *T. cruzi*-bearing cells were astrocytes located near IFNγ^+^ cells. Surprisingly, *in vitro* experiments revealed that pretreatment with IFNγ promoted the infection of astrocytes by *T. cruzi* increasing uptake and proliferation of intracellular forms, despite inducing increased production of nitric oxide (NO). Importantly, the effect of IFNγ on *T. cruzi* uptake and growth is completely blocked by the anti-tumor necrosis factor (TNF) antibody Infliximab and partially blocked by the inhibitor of nitric oxide synthesis L-NAME. These data support that IFNγ fuels astrocyte infection by *T. cruzi* and critically implicate IFNγ-stimulated *T. cruzi*-infected astrocytes as sources of TNF and NO, which may contribute to parasite persistence and CNS pathology in CD.

## Introduction

Chagas disease (CD), caused by the intracellular protozoan *Trypanosoma cruzi*, is an important public health problem in Latin America. In the last decade, CD has gained interest in non-endemic countries of Europe, United States of America and Japan as a result of human migration [1,2]. Although cardiac and intestinal pathologies are the most frequent and studied manifestations of this disease [2], the central nervous system (CNS) is also seriously compromised in CD as meningoencephalitis appears as an important cause of death in the acute phase of infection [3]. In chronic CD patients, the CNS is the primary site of severe lesions in episodes of reactivation in immunosuppressive conditions as human immunodeficiency virus (HIV) coinfection [4,5]. Further, chronically chagasic patients frequently present cognitive disturbs and behavioral alterations as sleep dysfunction, memory impairment and depression [2,3]. Recently, depressive-like behavior was reproduced in chronically *T. cruzi*-infected mice [6].

We have found that astrocytes are target cells during *in vivo* infections with the Colombian *T. cruzi* Type I strain, [6] and others have suggested that astrocytes are infected by *T. cruzi* regardless of strain [7]. Indeed, a recent study showed that the human astrocytoma cell line CRL-1718 is highly susceptible to *T. cruzi* infection [8]. Astrocytes are the most abundant glial cell type in the human CNS, constituting around 50% of the brain volume [9,10]. In the recent years, in addition to the classical support functions, maintenance of the CNS tissue homeostasis and participation in the blood-brain barrier formation, astrocytes were also shown to take part of synapse formation and plasticity, maintenance of cerebrovascular tone, adult neurogenesis, among other functions [9,11–13]. In situations of injury and/or infection, astrocytes are shown to be immunologically competent [14–16] and to respond to inflammatory and/or infectious stimuli by expressing a variety of immune system-related molecules such as class I and II major histocompatibility complex (MHC) antigens [17,18], chemokines and cytokines [19–21], complement factors [22] and nitric oxide (NO) [23,24]. Importantly, increased NO production leads to a state of oxidative stress, contributing to amplification of neurodegenerative processes in the CNS [25].

IFNγ is involved in control of parasite growth systemically and in the cardiac tissue [26,27]. It has been shown that IFNγ activates *T. cruzi*-infected macrophages and cardiomyocytes to produce reactive oxygen (ROS) and nitrogen (RNS) species, microbicidal molecules important for the control of intracellular amastigote forms of the parasite [28–31]. However, these canonical effects of IFNγ may not apply to the CNS during *T. cruzi* infection, where the presence of inflammatory cytokines is mostly detrimental [16]. Indeed, in experimental model of reactivation of *T. cruzi* infection during partial immunosuppression, behavioral abnormalities were associated with exacerbation of inflammation and parasitism in the CNS, even in the presence of IFNγ [32], suggesting a role for inflammation and IFNγ in *T. cruzi* dissemination in the CNS. Notably, in *in vitro* studies IFNγ promoted the infection of astrocytes by HIV [33,34]. Therefore, here we have questioned whether *in vivo* parasite persistence was associated with IFNγ expression and which effects IFNγ has on primary astrocyte cell cultures infected with the Colombian *T. cruzi* strain, evaluating susceptibility to infection and cell activation.

## Materials and Methods

### 2.1 Ethics statement

This study was carried out in strict accordance with the recommendations in the Guide for the Care and Use of Laboratory Animals of the Brazilian National Council of Animal Experimentation (http://www.cobea.org.br/) and the Federal Law 11.794 (October 8, 2008). The institutional Committee for Animal Ethics of Fiocruz (CEUA/Fiocruz, License 004/09) approved all the procedures used in the present study.

### 2.2 Animals, parasites and experimental *T. cruzi* infection

Four- to six-week-old female mice of the C3H/He (H-2^*k*^) lineage with an average weight of between 15–22 g were obtained from the animal facilities of the Oswaldo Cruz Foundation (CECAL, Rio de Janeiro, Brazil) and maintained in conditions of specific pathogen free. Mice were infected intraperitoneally with 100 blood trypomastigote forms of the Colombian *T. cruzi* Type I strain [35], which has previously been shown to colonize the CNS [36,37]. The parasite was maintained by serial passage in mice every 35 days post-infection (dpi). Parasitemia was quantitated weekly during the acute and chronic infection phases from 5 μL of tail vein blood, as previously described [27], and the presence of the rare trypomastigotes marked the onset of the chronic phase of infection, as described elsewhere [36,37]. Mice were sacrificed under anesthesia (100 mg/Kg ketamine associated with 5 mg/Kg xylazine chloride) at parasitemia peak (42 dpi; acute phase) and when parasitemia was controlled (90 dpi; chronic phase), as previously described [6]. The experimental groups were composed of six to ten *T. cruzi*-infected mice and three to five noninfected (NI) controls per experiment, in three independent experiments.

### 2.3 Immunohistochemical staining (IHS) for parasite and cytokine detection

Groups of six infected mice and three sex- and age-matched NI controls were sacrificed under anesthesia at 42 and 90 dpi. The encephalons were removed, embedded in tissue-freezing medium (Tissue-Tek, Miles Laboratories, USA) and stored in liquid nitrogen for analysis by IHS. Serial cryostat sections (3 μm thick) were fixed in cold acetone and subjected to immunofluorescence staining. Parasite antigens were stained with the polyclonal rabbit anti-*T. cruzi* antibody (produced in our laboratory, LBI/IOC-Fiocruz, Brazil) and FITC-labeled secondary goat anti-rabbit antibody (Amersham, England). Astrocytes were marked with purified rat anti-glial fibrillary acidic protein (GFAP) antibody (Invitrogen, USA), and biotin-conjugated anti-rat antibody (Dako, USA) plus streptavidin-APCCY7 complex (BD, USA). IFNγ was stained using PE-labelled anti-mouse IFNγ monoclonal antibody (BD, USA). For negative controls, brain tissue sections from infected mice were subjected to all the steps of the reaction omitting the primary antibodies. The images were acquired on a fluorescence microscope (Nikon Eclipse CI, Japan) and analyzed with the software Nis-Elements BR 4.0. For detection of parasites in the CNS tissue, the areas containing parasites were identified and the data shown as parasite positive sections per 10 sections analyzed for each brain. To study the spatial distribution of GFAP^+^ cells, *T. cruzi* antigens^+^ areas and IFNγ-producing cells, five whole brain sections were analyzed per each mouse. The data are shown as merged images of microphotographs and frequency of associated spots per brain section.

To identify the source of IFNγ in the CNS during *T. cruzi* infection, in a series of experiments serial cryostat sections (3 μm thick) were fixed in cold acetone and subjected to indirect immunoperoxidase staining. Each section was stained using: biotin-conjugated anti-CD3 (BD, USA) followed by streptavidin-peroxidase, to label T-cells; anti-Iba1 (Wako Chemicals, USA) followed by biotin-conjugated anti-rabbit IgG (Ge Healthcare, UK) and streptavidin-peroxidase, to reveal microglial cells; or anti-IFNγ (BD Biosciences, USA) followed by biotin-conjugated anti-rat (Dako, USA) and streptavidin-peroxidase. Serial sections were also stained for *T. cruzi* antigens using the polyclonal anti-*T. cruzi* antibody followed by biotin-conjugated anti-rabbit IgG and streptavidin-peroxidase. Additionally, serial sections were stained using anti-GFAP antibody (Dako, USA), biotin-conjugated anti-rat antibody (Dako, USA) and streptavidin-peroxidase complex (Ge Healthcare, UK), to reveal astrocytes. Additionally, we used spleen and heart tissue sections of acutely infected mice (25 and 42 dpi) as positive controls for *T. cruzi* antigens and cell markers, as described elsewhere [38].

### 2.4 RT-PCR assay for IFNγ mRNA

Animals were euthanized under anesthesia and submitted to perfusion with saline to wash out blood contamination. The whole encephalon was used to isolate mRNA by acid guanidinium thiocyanate-phenol-chloroform extraction. The RNA STAT-60 reverse transcriptase-PCR conditions, primer sequences used for the detection of IFNγ, housekeeping gene hypoxanthine-guanine phosphoribosyltransferase (HPRT) and PCR product sizes have been published elsewhere [38]. The PCR products and a molecular size marker were electrophoresed in 6% polyacrylamide gel and stained with silver nitrate. The densitometry analysis of the gels was conducted on a Densitometer CS-9301PC (Shimadzu, Japan). The PCR data were standardized using mRNA of the housekeeping gene HPRT and fold increases were determined by a comparison with NI controls.

### 2.5 Primary mouse astrocyte cell cultures

Primary mouse astrocyte cell cultures were obtained from the brain cortex of 1-day-old neonatal C3H/He (H-2^*k*^) mice as previously described [39]. In a set of experiments, 1-day-old neonatal C57BL/6 (H-2^*b*^) mice were also used. Briefly, mice were decapitated and meninges removed. Cortices were isolated, minced and a single cell suspension obtained by mechanical dissociation. The cells from each brain were seeded into a 25 cm^2^ culture flask precoated with poly-ornithine (0.1 mg/mL; Sigma, USA) in DMEM/F-12 medium (Gibco, USA) containing 10% fetal bovine serum (FBS; Gibco, USA) and maintained at 37°C kept in 95% humid atmosphere with 5% of CO_2_. Microglial and other non-adherent cells were removed by soft shaking the culture flasks. Astrocytes from seven to ten days of culture were detached with trypsin 0.125% EDTA and plated at a density of 10^5^ cells per 13 mm diameter poly-ornithine-coated coverslip in 24-well plates (NUNC, Denmark). Cells were kept 24-hours in culture for to allow adhesion of astrocytes to coverslips. Details of each experiment are described in the figure legends.

### 2.6 Characterization of primary mouse astrocyte cell cultures

For this purpose, we performed indirect immunofluorescence using purified mouse anti-GFAP (clone 2.2B10, Invitrogen, USA) and FITC-conjugated secondary antibody anti-mouse immunoglobulin (Dako, USA). Microglia was revealed by the surface expression of CD11b molecule using PECy7-conjugated monoclonal anti-mouse CD11b (clone M1/70 eBioscience, USA). After cultivation, cells were washed in phosphate buffered saline (PBS) pH 7.2–7.4 at 37°C and fixed with methanol. To block non-specific binding, the cells were incubated for 30 min with normal goat serum diluted 1:50 in PBS 0.1% sodium azide and then stained for GFAP and CD11b. Negative control was done by omitting the primary antibodies and using only the secondary antibody. The slides were mounted with prolong gold antifade reagent with DAPI (Life Technologies, USA) and examined and photo documented using a fluorescence microscope (Leica DFC300FX, Germany). The images were analyzed using the software IM50 Image Manager (Leica, Germany).

### 2.7 Fibroblast cell line

The fibroblast cell line L-929 (ATCC, CCL-1), originated from a C3H mouse, was kindly provided by Dr. Maria de Nazaré Correia Soeiro (Laboratory of Cellular Biology, IOC, Fiocruz). Cells were cultivated in RMPI medium supplemented with 10% FBS and 1% penicillin-streptomycin (Sigma, USA) and maintained at 37°C in 95% humid atmosphere with 5% CO_2_. Cells were detached with trypsin 0.125% EDTA and plated at a density of 5×10^4^ cells per 13 mm of diameter poly-ornithine-coated coverslip in 24-well plates (NUNC, Denmark). Cells were kept 48-hours in culture to allow adhesion to coverslips. Details of each experiment are described in the figure legends.

### 2.8 Parasites for *in vitro* studies

Trypomastigote forms of the Colombian *T. cruzi* strain were obtained from cultures of Vero cell line (CCL81, ATCC, USA), kept in cultivation bottles of 150 cm^2^ (Falcon, USA), using DMEM medium supplemented with 10% FBS (Sigma-Aldrich, USA). After seven days of culture, the trypomastigote-containing supernatants were centrifuged at low speed (120 x *g*) for 15 min to remove cell debris. The supernatant was transferred to another tube and high-speed centrifugation (1700 x *g* at 4°C) for 15 min was used to pellet the trypomastigotes. Parasites were resuspended, counted in a Neubauer chamber using Trypan, blue for exclusion of dead parasites, and used when viability was > 95%.

### 2.9 Treatment of astrocytes and fibroblasts with IFNγ and *Trypanosoma cruzi* infection

In order to assess the effects of treatment with IFNγ on infection of astrocytes by *T. cruzi*, astrocytes were plated at density of 10^5^ cells per 13 mm of diameter poly-ornithine-coated coverslip in 24-well plates and allowed to adhere. After 24-hours, cultures were washed three times with PBS at 37°C to remove non-adherent cells, medium was replaced and astrocytes were pretreated or not with 10 ng/mL of murine recombinant IFNγ (eBioscience, USA) for 2 hours before infection with trypomastigotes of the Colombian *T. cruzi* strain at a multiplicity of infection (MOI) 1 or 10 parasites per astrocyte. In a set of experiments, IFNγ was added after the infection according to the experimental schemes shown in the figures.

To assess the effects of IFNγ in cells other than astrocytes, we also infected the murine fibroblast cell line L-929 with *T. cruzi* in either in presence or absence of IFNγ. Cells were plated as described above and allowed to adhere. Afterwards, cultures were washed with PBS and the medium was replaced. Fibroblasts were either treated or not with IFNγ (10 ng/mL) for 2 hours and then infected with parasites, essentially as described for astrocytes.

The plates were incubated at 37°C in environment with 95% humidity and 5% CO_2_ during periods of interest in each experiment. Then, the cell cultures were washed three times with PBS at 37°C and fixed in Bouin fixative (picric acid-formalin-acetic acid mixture) overnight at room temperature. After three washing steps with PBS, the coverslips were stained for 3 hours with Giemsa (Merck, Germany), dehydrated in acetone/xylol and mounted in Entellan (Merck, Germany). The numbers of infected cells and of intracellular forms of *T. cruzi* per cell were assessed using a light microscopy (Nikon, USA).

### 2.10 Evaluation of TNF participation in the increased IFNγ-induced *T. cruzi* infection of astrocytes

In an attempt to evaluate the role of TNF in the infection of astrocytes induced by IFNγ, we treated cells with the chimeric monoclonal antibody Infliximab (10 μg/mL; Remicade, Schering-Plough, Brazil) 30 min before adding IFNγ. Two hours after IFNγ treatment, cells were infected with parasites for 4 hours, washed, fixed, stained and coverslips mounted as described above.

### 2.11 Evaluation of NO inhibition in the increased IFNγ-induced *T. cruzi* infection of astrocytes

To evaluate the role of NO in IFNγ-induced *T. cruzi* infection of astrocytes, cells were treated with 1 mM L-NAME hydrochloride (Sigma, USA), an arginine analog that inhibit NO production, 30 min before adding IFNγ. Two hours after IFNγ treatment, cells were infected with *T. cruzi* for 24 hours, washed, fixed, stained and mounted as described above.

### 2.12 Treatment of astrocytes with IFNγ and LPS and evaluation of NO production

To test the ability of primary mouse astrocyte cell cultures to produce NO, cells were prepared as described above, treated with IFNγ (10 ng/mL), LPS derived from *Escherichia coli* (Sigma, USA) (10 ng/mL), IFNγ plus LPS (LPS was added 2 hours after IFNγ treatment) or left untreated (NT). Supernatants were collected after 24- and 48-hours. Additionally, cells of the RAW 264.7 mouse macrophage cell line (TIB-71, ATCC, USA) were cultured in parallel and treated with IFNγ, LPS or IFNγ plus LPS as positive controls. Nitric oxide (NO) production was assessed by Griess reaction [40]. The sample concentrations were estimated based on a standard curve from 100 to 0.8 μM sodium nitrite. The efficacy of L-NAME on inhibiting NO production was also assessed on RAW 264.7 macrophages in a set of experiments.

### 2.13 Statistical analysis

Data are expressed as arithmetic means ± SEM. Comparison between groups was carried out by unpaired t-Student test or one-way ANOVA followed by Bonferroni test, when appropriate, using GraphPad Prism Software. Differences were considered statistically significant when *p<*0.05.

## Results

### 3.1 *Trypanosoma cruzi* persists in the CNS inside astrocytes

**Fig 1:**
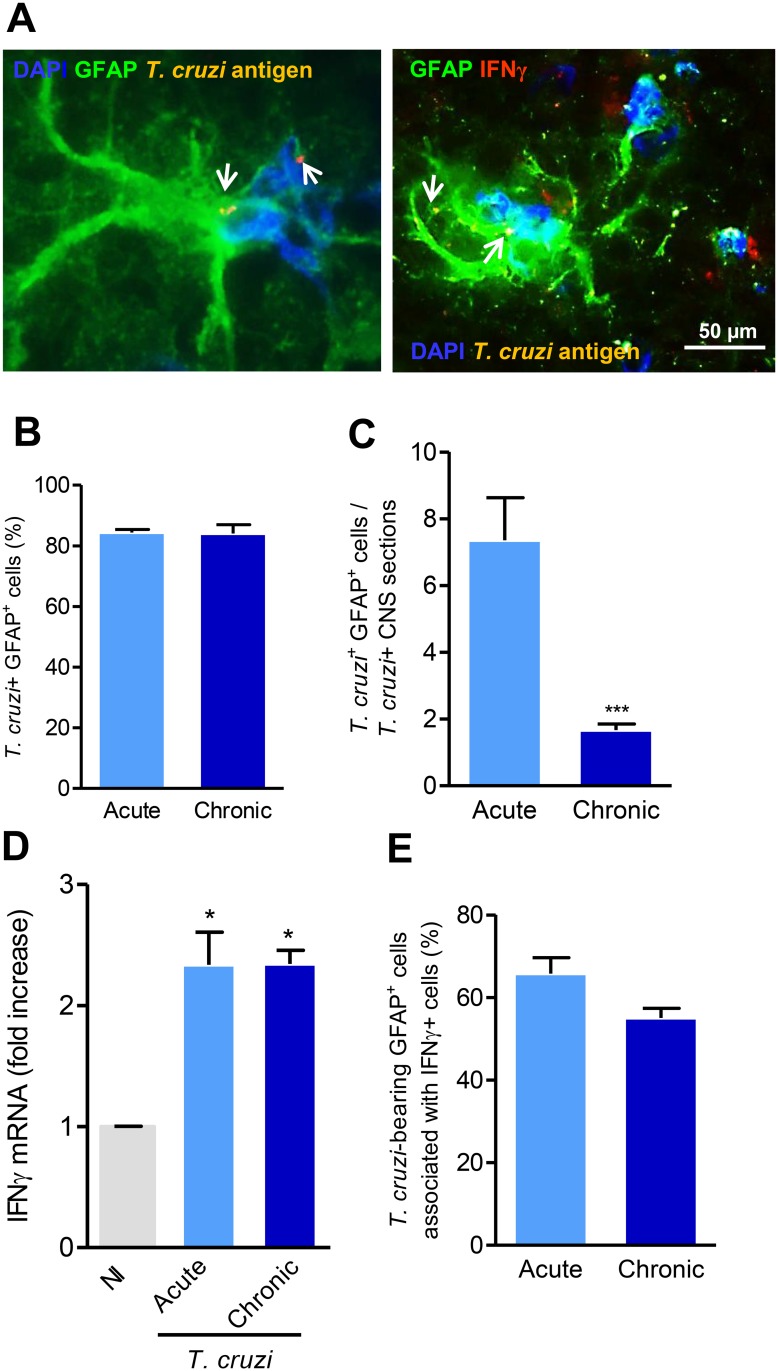
*Trypanosoma cruzi* antigens are detected in the central nervous system inside astrocytes in the presence of IFNγ. (A) Immunohistochemical staining of CNS sections of acutely (42 dpi) *T. cruzi*-infected mice showing parasite antigens-bearing astrocytes (left panel) and parasite antigens-bearing astrocyte surrounded by IFNγ-expressing cells (right panel). Arrows indicate parasite antigens detected by immunohistochemical staining using polyclonal anti-*T. cruzi* antibody. (B) Frequency of *T. cruzi* antigens^+^ GFAP^+^ cells among *T. cruzi* ^+^ areas in the CNS of acute (42 dpi) and chronically (90 dpi) infected mice. (C) Number of *T. cruzi* antigens^+^ GFAP^+^ cells per *T. cruzi* ^+^ CNS section in acute and chronically infected mice. (D) IFNγ mRNA expression in the CNS of *T. cruzi*-infected C3H/He mice compared with noninfected (NI) controls. (E) Frequency of *T. cruzi* antigens^+^ GFAP^+^ cells associated with IFNγ^+^ cells in the CNS of acute and chronically infected mice. The experimental groups were composed of six infected mice and three NI sex- and age-matched control mice. *, *p* < 0.05, ***, *p* < 0.001.

When infected with the Colombian *T. cruzi* Type I strain, C3H/He mice showed peak of parasitemia at 42 dpi (acute phase). Parasites in the blood decreased thereafter and most of the animals showed negative parasitemia by 90 dpi, corroborating previous data [6]. Interestingly, despite most animals presented negative parasitemia during chronic phase, the immunohistochemical analysis of ten CNS serial sections per mouse showed that 100% of mice during acute phase and 67% of chronically infected mice presented *T. cruzi* ^+^areas in the CNS. CNS parasitism was mainly detected as amastigote forms of the parasite, 80% of the *T. cruzi* ^+^ area coincided with GFAP^+^ cells (astrocytes) spread throughout brain parenchyma (Fig. 1A, B) in both, acute and chronic phases. Compared with acute infection, a reduced number of *T. cruzi*-bearing GFAP^+^ cells was observed in the CNS during the chronic phase of infection (Fig. 1C).

### 3.2 *Trypanosoma cruzi* persists inside astrocytes in the presence of IFNγ

To investigate whether IFNγ plays a role in *T. cruzi* dissemination in the CNS, we analyzed IFNγ mRNA expression in the encephalon of NI controls and *T. cruzi*-infected mice. In comparison with NI controls, a significant increase in IFNγ mRNA expression was detected in the CNS of C3H/He mice in the acute and chronic phases of infection by the Colombian *T. cruzi* strain (Fig. 1D), suggesting that parasite persists in the brain even in the presence of significant IFNγ mRNA expression.

The study of serial sections of the CNS showed that GFAP^+^ cells coincided with *T. cruzi* antigen^+^ areas and IFNγ^+^ cells (S1A Fig.). Additionally, the co-staining of brain sections for parasite antigens with anti-*T. cruzi* antibody (yellow), astrocytes cells with anti-GFAP antibody (green), IFNγ^+^ cells with PE-labelled anti-IFNγ (red) and nuclei revealed by DAPI (blue) showed that frequently the *T. cruzi*-bearing GFAP^+^ cells were seen in very close proximity to IFNγ^+^ cells (Fig. 1A, E) during both acute (67 ± 6.7%) and chronic (55 ± 4.0%) phases of the infection. In previous work, we have shown that C3H/He mice are susceptible to acute phase-restricted meningoencephalitis and rare CD4^+^ and CD8^+^ cells are detected in the CNS of chronically infected mice [37]. T-cells and NK cells are important sources of IFNγ [41], but this cytokine may also be produced by microglial cells in the CNS [42,43]. To study the potential sources of IFNγ in the CNS of *T. cruzi*-infected mice, serial sections of the CNS were analyzed by indirect immunohistochemistry to amplify staining using anti-CD3, anti-Iba1 and anti-IFNγ antibodies. Our data support that IFNγ^+^ cells are matched to Iba1^+^ cells (microglial cells), but not with CD3-expressing cells (T-cells). CD3^+^ cells were identified in other areas of the CNS as well as spleen and heart tissue sections used as positive controls; therefore Iba1^+^ cells are potential sources of IFNγ in the CNS of *T. cruzi*-infected mice (S1B Fig.).

### 3.3 Astrocytes of mouse CNS primary cell cultures are targets of *Trypanosoma cruzi* infection

**Fig 2:**
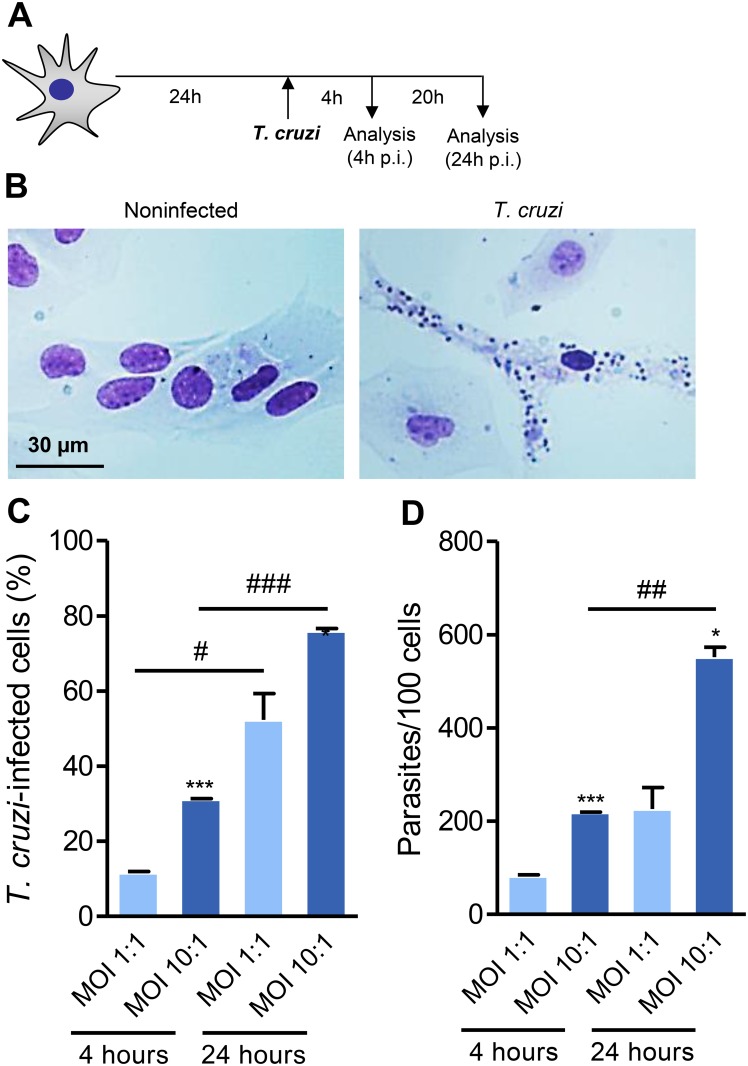
Astrocytes of murine CNS primary cultures are targets of *Trypanosoma cruzi* infection. (A) Experimental scheme showing that cultures of astrocytes were infected at MOI of 1:1 or 10:1 and analyzed 4 and 24 hours post-infection (p.i.). (B) Representative pictures of noninfected and *T. cruzi*-infected astrocytes at 4 hours of infection. (C) The percentage of infected cells (D) and the number of parasites per cells were analyzed. Data are presented as mean ± SEM of triplicates. *, *p* < 0.05 and ***, *p* < 0.001comparing MOI 1:1 with MOI 10:1 in each analyzed intervals; ^#^, *p* < 0.05, ^##^, *p* < 0.01 and ^###^, *p* < 0.001 comparing 4 hours with 24 hours in each studied MOI.

It is intriguing that CNS parasitism was associated to the presence of IFNγ-producing cells. To approach the effects of IFNγ on astrocytes submitted to *T. cruzi* infection we established an *in vitro* infection model of monotypic primary astrocyte cell cultures. After seven to ten days of culture, cells were plated on coverslips in 24-well plates. Morphological assessments were performed 24 hours later, when the majority of the cells showed thin extensions and delicate branches with cytoplasm lightly stained by Giemsa and recognizable nucleoli, morphologically resembling astrocytes (S2A, B Fig.). Because microglial cells are the most frequent contaminants of primary astrocyte cell cultures, to verify the cell composition of our primary cultures they were simultaneously labeled with antibody recognizing GFAP, the major protein of intermediate filaments in differentiated fibrous and protoplasmic astrocytes [16,44], and with antibody specific to the surface molecule of microglial cells CD11b [45]. We found a purity of 99% of GFAP^+^ (FITC, green) cells, while rare CD11b^+^ cells (PE, red) were detected (< 1%) in our cell cultures (S2C Fig.). Primary astrocyte cell cultures were infected with Colombian *T. cruzi* strain for 4 or 24 hours, as schematized (Fig. 2A). *In vitro*, mouse astrocyte cells were infected by trypomastigote forms and supported the differentiation to intracellular (amastigote-like) forms of *T. cruzi* (Fig. 2B). We used MOI of 1 and 10 to determine the frequency of infected cells at 4 hours (to assess rate of infection) and 24 hours (to estimate parasite proliferation) in astrocytes. The frequency of astrocytes bearing intracellular parasites is critically influenced by the time of parasite/astrocyte interaction (Fig. 2C). Also, the mean number of parasites per cell was determined by the MOI and the time of parasite/cell interaction (Fig. 2D).

### 3.4 Pretreatment of astrocytes with IFNγ enhances infection by *Trypanosoma cruzi*

**Fig 3:**
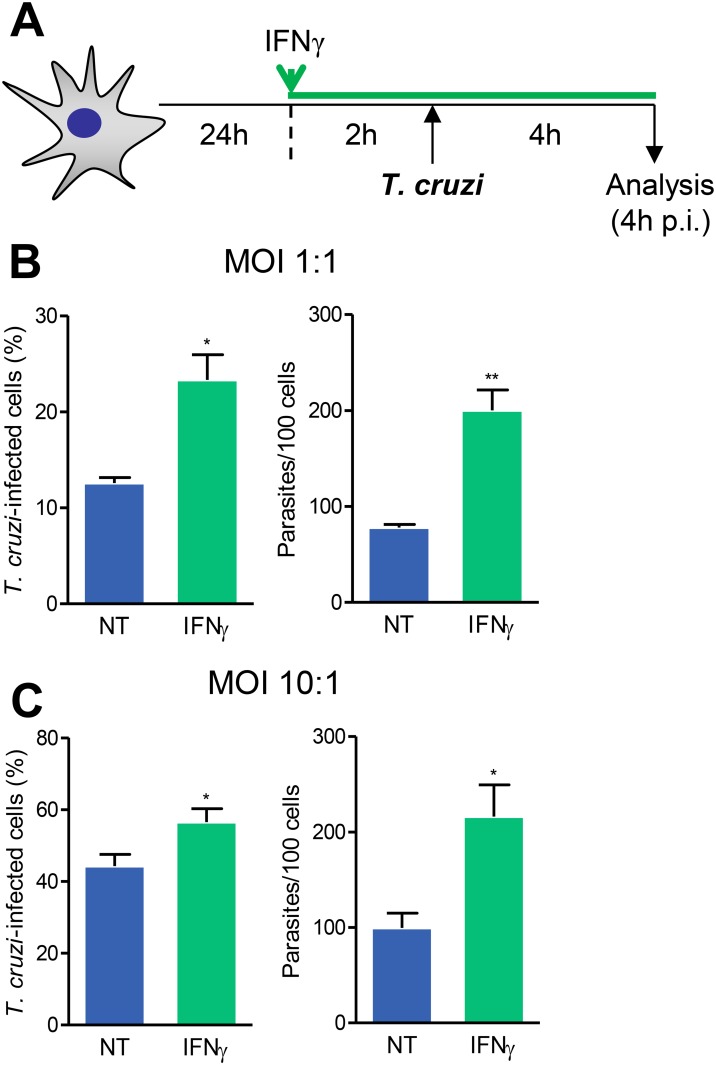
Pretreatment with IFNγ promotes astrocytes infection by *T. cruzi*. (A) Experimental scheme showing that primary astrocyte cell cultures were pretreated with IFNγ (10 ng/mL) for 2 hours and subsequently infected using MOI 1:1 or 10:1 and analyzed 4 hours post-infection (p.i.). (B) and (C) show the frequencies of *T. cruzi*-infected cells and the number of parasites per cells, when initial infection MOI was 1:1 or 10:1, respectively. Data are presented as mean ± SEM of triplicates. *, *p* < 0.05 and **, *p* < 0.01 comparing not-treated (NT) with IFNγ pretreated astrocytes in each studied MOI.

IFNγ influences *T. cruzi* replication in mouse and rat cardiomyocytes [30,46]. We, then, asked whether IFNγ interferes with *T. cruzi*/astrocyte interaction. Astrocytes were pretreated with IFNγ for 2 hours before infection with *T. cruzi* and parasite entry was measured 4 hours after infection (Fig. 3A). IFNγ-pretreated cultures infected at MOI of 1 and 10 showed increased frequency of infected cells and higher mean number of parasites per infected cells compared with not treated (NT) infected cultures (Fig. 3B, C).

Next, we tested whether the effect of pretreatment with IFNγ promoting *T. cruzi*/astrocyte interaction was a common biological process in other cell types. To challenge this idea, we pretreated L-929 mouse fibroblasts with IFNγ and infected with *T. cruzi* (S3A Fig.). Pretreatment of L-929 fibroblasts with IFNγ had no effect on *T. cruzi*/fibroblast interaction, regardless the MOI used (1:1 or 10:1) and the time (4 or 24 hours) of *T. cruzi*/fibroblast interaction (S3B-E Fig.).

### 3.5 IFNγ promotes amastigote growth in *T. cruzi*-infected astrocytes

**Fig 4:**
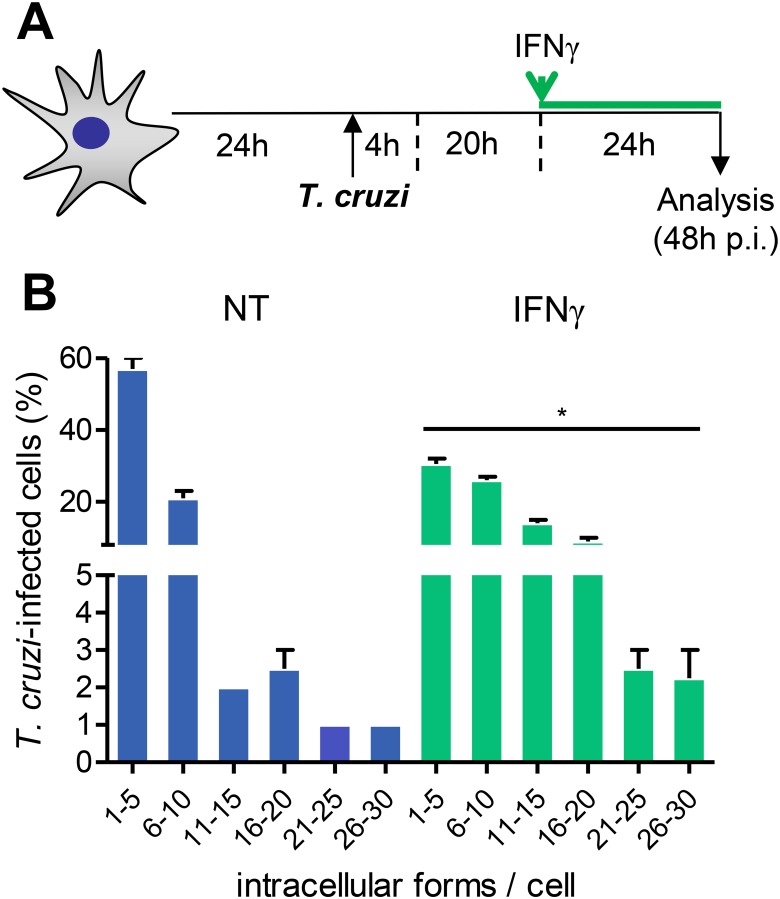
Treatment of amastigote-bearing astrocytes with IFNγ enhances parasite growth. (A) Primary astrocyte cell cultures were infected by *T. cruzi* (MOI 1:1) for 4 hours. Afterwards, parasites were washed out, culture medium was replaced and cells were incubated for an additional 20 hours. Amastigote-bearing astrocytes were treated or not with IFNγ (10 ng/mL) and analyzed 24 hours later (48 hours post-infection, p.i.). (B) The graph shows the frequencies of *T. cruzi*-infected cells bearing different classes of parasite load. Data are presented as mean ± SEM of triplicates. *, *p* < 0.05 comparing not-treated (NT) with IFNγ-treated astrocytes.

To test whether IFNγ plays a role on parasite growth inside astrocytes, we submitted astrocytes carrying *T. cruzi* amastigote forms to IFNγ treatment and analyzed 24 hours later, as schematized (Fig. 4A). Interestingly, the treatment of *T. cruzi*-infected astrocytes with IFNγ increased the frequency of amastigote-bearing astrocytes and the numbers of parasites per cell compared with NT astrocytes (Fig. 4B). Moreover, IFNγ increased the frequency of astrocytes carrying large numbers of amastigote forms (over 10 parasites per cell) in their cytoplasm (Fig. 4B). Interestingly, IFNγ also promoted amastigote growth inside astrocytes of the C57BL/6 mouse strain (S4 Fig.), supporting that this was a conserved biological effect of this cytokine during chagasic infection.

### 3.6 Two signals are required for nitric oxide production by *T. cruzi*-infected mouse astrocytes

**Fig 5:**
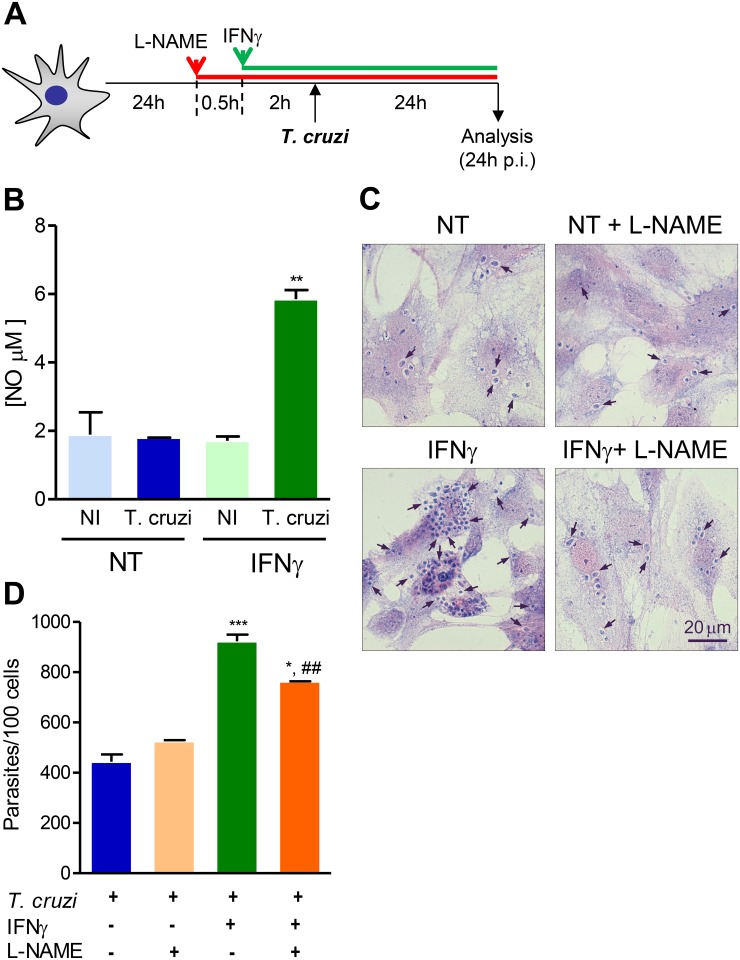
Two signals are required for nitric oxide production by *T. cruzi*-infected astrocytes. (A) Primary astrocyte cell cultures were pretreated or not with IFNγ (10 ng/mL) in the absence or presence of L-NAME, infected using MOI 10:1 and analyzed 24 hours post-infection (p.i.). (B) The graph shows NO production by astrocytes treated or not (NT) with IFNγ and infected or not (NI) with *T. cruzi*. (C) Representative pictures of IFNγ- pretreated astrocytes infected in the absence or presence of L-NAME. (D) The graph shows the number of intracellular forms of the parasite per 100 cells in the different experimental conditions. Data are presented as mean ± SEM of triplicates. *, *p* < 0.05, **, *p* < 0.01 and ***, *p* < 0.001 comparing IFNγ pretreated astrocytes with NT cells; ^##^, *p* < 0.01 comparing IFNγ pretreated astrocytes in absence or presence of L-NAME.

IFNγ is a potent inducer of NO production by cardiomyocytes under *T. cruzi* infection [30] and astrocytes can produce NO when stimulated with LPS and IL-1β [47]. We then evaluated the effect of *T. cruzi* infection and exposition to IFNγ on NO production by astrocytes (Fig. 5A). Neither IFNγ nor *T. cruzi* infection alone were able to induce NO production by astrocytes (Fig. 5B) in the experimental conditions herein described. On the other hand, the same IFNγ concentration (10 ng/mL) induced NO production by the mouse macrophage cell line RAW 264.7 (S5A Fig.). Contrasting to macrophages, which promptly produced NO after treatment with IFNγ (10 ng/mL) and/or the TLR-4 agonist LPS (10 ng/mL) (S5B Fig.), astrocytes of primary cell cultures required two signals (IFNγ plus LPS) for full stimulation and NO production (S5C Fig.). In the case of *T. cruzi* infection, astrocytes required two signals (IFNγ treatment and *T. cruzi* infection) for production of significantly detectable levels of NO (Fig. 5B).

### 3.7 Effect of IFNγ promoting *T. cruzi*/astrocyte interaction depends partially on NO

Intense parasitism of CNS paralleled NO overproduction in IL-12-deficient *T. cruzi*-infected mice [48] and NO is produced by IFNγ-pretreated astrocytes infected by *T. cruzi* (Fig. 5B). Thus, we tested the participation of NO on IFNγ-pretreated induction of astrocyte infection by *T. cruzi*. The efficacy of L-NAME hydrochloride, an inhibitor of NO production was confirmed on IFNγ/LPS-stimulated RAW 264.7 macrophages (S5D Fig.). Interestingly, addition of L-NAME to IFNγ-pretreated astrocytes partially reduced (*p*<0.01) the number of amastigote forms of *T. cruzi* inside astrocytes (Fig. 5C, D).

### 3.8 IFNγ-enhanced astrocyte infection by *T. cruzi* is prevented by TNF blockade

**Fig 6:**
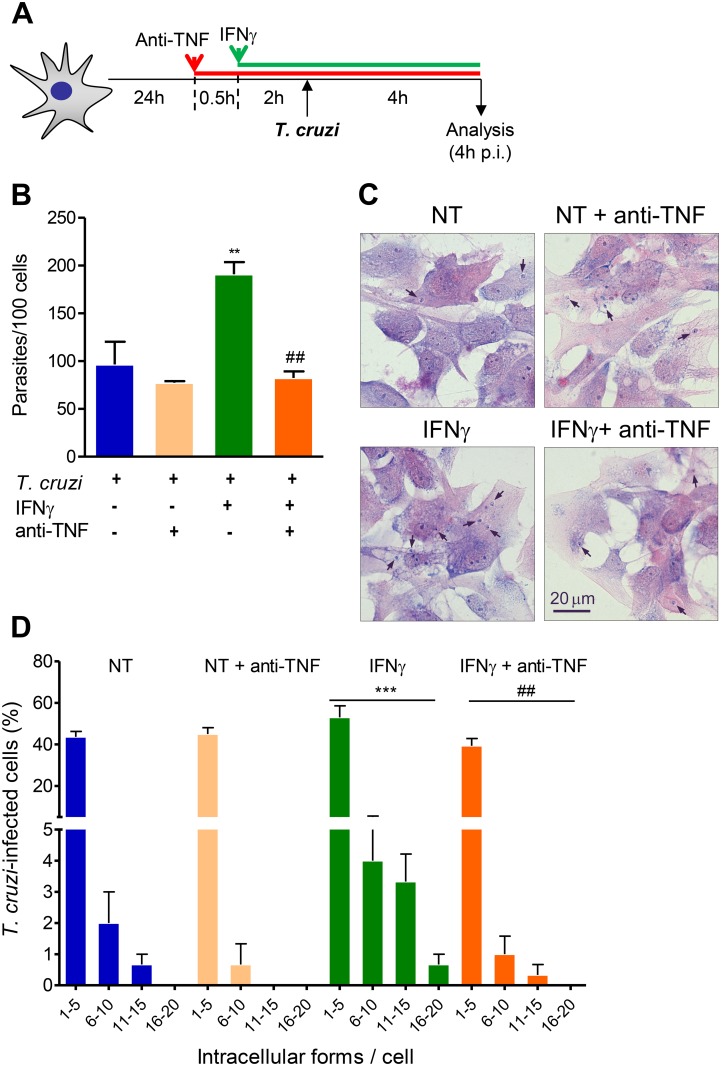
Anti-TNF antibody prevented the effects of IFNγ on infection of astrocytes by *T. cruzi*. (A) Primary astrocyte cell cultures were treated or not with IFNγ (10 ng/mL) in the absence or presence of anti-TNF (10 μg/mL), infected using MOI 10:1, and analyzed 4 hours post-infection (p.i.). (B) Graph shows the mean number of parasites per 100 cells. (C) Representative pictures of IFNγ pretreated astrocytes and infected in the absence or presence of anti-TNF. (D) The graph shows the frequencies of *T. cruzi*-infected cells bearing different numbers of intracellular forms of the parasite. Data are presented as mean ± SEM of triplicates. **, *p* < 0.01 and ***, *p* < 0.001 comparing IFNγ-pretreated astrocytes with not-treated (NT) cells; ^##^, *p* < 0.01 comparing IFNγ pretreated astrocytes in absence or presence of anti-TNF.

Astrocytes are sources of cytokines, which are upregulated by IFNγ [16]. Therefore, we tested whether IFNγ directly enhanced astrocytes infection by *T. cruzi* or whether this effect depends on TNF induction. For that purpose, IFNγ-pretreated astrocytes were infected with *T. cruzi* in the presence of the TNF neutralizing antibody Infliximab (Fig. 6A). Notably, similar numbers of intracellular parasites were detected in NT *T. cruzi*-infected astrocytes and IFNγ-pretreated astrocytes incubated with Infliximab (Fig. 6B). Importantly, anti-TNF abolished the effect of IFNγ-induced increase of the frequency of cells bearing high numbers of intracellular forms of the parasite (Fig. 6C, D). Indeed, anti-TNF completely prevented the IFNγ-enhanced infection of astrocytes by *T. cruzi* (Fig. 6B-D).

## Discussion

In the present work, we found that in C3H/He mice infected by the Colombian *T. cruzi* strain parasite persistence, mainly inside astrocytes, in the CNS was associated with expression of IFNγ mRNA. Further, *T. cruzi*-infected astrocytes were frequently in close relation to IFNγ^+^ cells. Therefore, we investigated the participation of IFNγ in astrocyte infection by *T. cruzi* and found that this cytokine promoted parasite uptake and growth in primary astrocyte cell cultures. Dual signals (*T. cruzi* infection and IFNγ) were required to induce NO production by astrocytes. Further, NO played a role in IFNγ-induced infection of astrocytes by *T. cruzi*. Moreover, IFNγ-induced effect on astrocyte infection was totally abrogated by the anti-TNF antibody Infliximab.

CNS is an important site for *T. cruzi* persistence in the host during the chronic phase of infection [32,37]. Not surprisingly, we detected intense parasitism in the CNS tissue of C3H/He mice infected by the Colombian *T. cruzi* Type I strain in the acute phase of infection followed by a drastic reduction of CNS parasitism in chronic phase. The majority of the infected mice showed CNS parasitism detectable only by immunohistochemical staining, corroborating previous data suggesting that despite being an immune specialized site, the CNS is able to control, but not eliminate, tissue parasitism [6,36,37]. Glial cells of the CNS were firstly shown to be targets of *T. cruzi* infection in an experimental dog model of CD [49]. In previous works, we showed that in acutely-infected C3H/He mice, astrocytes (GFAP^+^ cells) and macrophages/microglial cells (F4/80^+^) are targets of infection [6]. Furthermore, in rats infected with Y or CL-Brener clone *T. cruzi* [7], Type II and Type VI (hybrid II/III genotypes) strains respectively [35], the majority of *T. cruzi*-infected cells morphologically resembled astrocytes. Here we confirmed that in the CNS of acute and chronically Colombian-infected C3H/He mice, the majority of *T. cruzi*-infected cells are GFAP^+^, therefore, astrocytes. Altogether, these data support that astrocytes are the main targets of *T. cruzi* infection in the CNS independently of the host specie and the parasite genotype.

Chronically CD patients coinfected with HIV show neurological abnormalities associated with massive *T. cruzi* parasitism in the CNS [3,4]. Importantly, the neurological abnormalities seen in CD patients coinfected with HIV can be successfully treated using the trypanocidal drug benznidazole, supporting parasite contribution to CNS impairment [3]. Although it was not clear whether the parasites present in the CNS in reactivation episodes were originated from *T. cruzi* forms in the CNS cells, cerebrospinal fluid or if they are blood-born parasites, the reactivation of *T. cruzi* infection in chronically infected C3H/He mice submitted to immunosuppressive therapy occurred in the absence of parasitemia and was restricted to the CNS [36]. Indeed, we found *T. cruzi* antigen in the CNS of chronically infected mice, especially inside astrocytes, not only suggesting that parasite persists in the CNS during chronic phase of infection, but also implicating astrocytes in parasite persistence. Interestingly, we found infected astrocytes in close association with IFNγ-producing cells.

The role of IFNγ in CD is well-known. Infection by *T. cruzi* triggers a strong proinflammatory response, especially composed of IFNγ, a cytokine associated with infection control [26] both systemically [50,51] and in the heart tissue [27,38]. Indeed, *T. cruzi* amastigote replication and trypomastigote release were significantly reduced in IFNγ-treated *T. cruzi*-infected primary macrophage and cardiomyocyte cell cultures [30,52]. However, the effects of IFNγ in the CNS in the presence of *T. cruzi* infection remained unexplored. Interestingly, mice which are partially, but not entirely, deficient in IFNγ display massive CNS *T. cruzi* parasitism. For example, IL-12-deficient mice, which can produce small levels of IFNγ, are able to partially control systemic infection, but display an even more dramatic CNS parasitism than IFNγ-deficient mice [32]. This finding suggests a role for inflammatory factors, such as IFNγ, in *T. cruzi* parasitism in the CNS.

Here we demonstrated that parasite persisted in the presence of IFNγ mRNA expression and production in the CNS in acute and chronic *T. cruzi* phases of infection. Although intense CD8-enriched meningoencephalitis is restricted to acute *T. cruzi* infection [36,37], few inflammatory lymphocytes were detected in the CNS of chronically infected C3H/He mice [36,37]. Therefore, these cells were initially assumed as intrinsical source of the IFNγ mRNA detected in the CNS [13]. Indeed, few IFNγ^+^ mononuclear cells were detected in the CNS of chronically infected mice, frequently associated with *T. cruzi*-bearing GFAP^+^ cells in this tissue. Additionally, IFNγ is also produced by microglial cells in the CNS during infectious and neurodegenerative diseases [42,43]. Indeed, our data support that microglial cells may contribute to IFNγ production in the CNS in chagasic infection. However, we cannot exclude the possibility that IFNγ represents a fingerprint associated with the release of other effector molecules or cytokines. Nevertheless, IFNγ^+^ cells, either by producing IFNγ or any other effector molecules and/or by other mechanisms, may play a dual role in the CNS, shutting down the high parasite burden in the acute infection, while fueling low parasitism in the chronic phase of infection. Therefore, we raised the idea that IFNγ, by acting on astrocytes, may contribute to the CNS colonization by *T. cruzi*. Interestingly, pretreatment of L-929 mouse fibroblasts with IFNγ had no effect on infection by *T. cruzi*. This finding contrasts with the inhibitory effect of IFNγ on *T. cruzi* replication in 3T3 mouse fibroblast [53], but corroborates the absence of effect of IFNγ in *T. cruzi*-infected human skin fibroblasts [54]. Altogether, these data support that the effect of IFNγ on astrocyte infection by *T. cruzi* is not a common biological process, highlighting the importance of the findings herein unveiled.

Recently, the CRL-1718 human astrocyte cell line (astrocytoma) was shown to be highly susceptible to *T. cruzi* infection [8]. Interestingly, the infection of this astrocyte cell line by *T. cruzi* upregulated class I and II MHC antigens, suggesting that *T. cruzi*-infected astrocytes may interact with T-cells and act as antigen presenting cell [8]. Whether or not such interaction is important for protective anti-*T. cruzi* immunity remains to be determined. Conversely, infection of this same astrocytoma cell line by *T. cruzi* down-modulated the expression of CCL2/JE/MCP-1, a CC-chemokine shown to control *T. cruzi* growth in primary cultures of macrophages [52] and cardiomyocytes [30]. Further, CCL2 controls parasite burden, cell infiltration and mononuclear cell activation in the acute *T. cruzi* infection [55].Therefore, in the capacity of shutting down this important effector pathway of the protective immune response may reside *T. cruzi* persistence, mainly in astrocytes, in the CNS of chronically infected mice. This idea deserves further exploration.

Astrocytes, the most abundant cells in the CNS, perform various biological functions, including those related to neuro-immune-inflammatory context as in situations of injury and/or infection [16,56–58]. There is a gap of knowledgement of the effects of inflammatory products on the response of astrocytes to infection with pathogens as the protozoan parasite *T. cruzi*. Here we tested the ability of astrocyte to produce NO under influence of priming with IFNγ and *T. cruzi* infection. Nitric oxide is an inflammatory mediator promptly induced by *T. cruzi* infection that takes part of parasite replication control in macrophages and cardiomyocytes [30,52]. Notably, astrocytes primed with IFNγ or infected with *T. cruzi* did not produce detectable levels of NO. On the other hand, this low IFNγ concentration significantly induced NO production by the RAW macrophage cell line. We found that astrocytes of primary cell cultures required two signals (IFNγ and LPS) to produce significant levels of NO, supporting that these cells are able to produce NO when properly activated, as previously suggested [59]. Importantly, the dual signaling by IFNγ priming and *T. cruzi* infection, the condition that significantly increased infection rates and replication, also provided the conditions to trigger NO production by astrocytes. Although NO detected levels in the supernatant of IFNγ-primed *T. cruzi*-infected astrocytes were found to be low (5–7 μM) when compared to those produced by macrophages (25–30 μM), it is conceivable to propose that locally produced NO may contribute to fuel parasite growth [59–61]. Truly, addition of the NO inhibitor L-NAME to IFNγ-primed *T. cruzi*-infected astrocytes partially abrogated *T. cruzi* infection/growth inside astrocytes. In *T. cruzi*-infected IL-12P40 knockout mice, intense CNS parasitism paralleled NO overproduction. Further, in IL-12 absence IFNγ and S10003B0032 may contribute to inducible NO synthase activation and NO overproduction, which may contribute to neuronal cell death [48]. Therefore, we proposed that the sustained production of NO by *T. cruzi*-infected astrocytes is sufficient to maintain an inflammatory environment, which may contribute to parasite growth and CNS damage [62].

Although it is the first time that a cytokine, particularly IFNγ, is shown to promote *T. cruzi* infection and growth in astrocytes, other cytokines and cytokine-signaling pathways have been previously shown to promote *T. cruzi* cell invasion and growth inside other cells. Transforming growth factor-β receptors I and II take part in *T. cruzi* invasion and replication in epithelial cells [63]. Additionally, TNF-mediated NF-κB activation facilitates cellular invasion of non-professional phagocytic epithelial cell line by *T. cruzi* [64]. Astrocytes produce TNF, which is upregulated by IFNγ [16,65]. Therefore, we tested whether IFNγ promotes *T. cruzi* invasion in astrocytes directly or via TNF. The anti-TNF antibody Infliximab completely abrogated IFNγ effects on astrocyte infection by *T. cruzi*. Therefore, this finding opens a new pathway to be explored to unveil the mechanisms controlling *T. cruzi* persistence in the CNS, particularly considering the persistence of high TNF levels in the serum of chronic CD patients [50] and chronic experimental CD [51]. The biological mechanism by which IFNγ promotes infection and *T. cruzi* growth inside astrocytes was not established. Interestingly, the controversy whether astrocytes could support HIV infection was clarified by the demonstration that cytokines, especially IFNγ, facilitate substantial productive HIV infection of the U87MG astroglioma cell line and human fetal astrocytes [33]. Further, IFNγ induced HIV replication in the U87MG cell line by down-modulating pathways related to virus replication control, in a STAT3-dependent manner [65]. Altogether, these data support that IFNγ may crucially take part in the pathogenesis of diseases caused by different pathogens as viruses and protozoan parasites. More importantly, IFNγ may have a crucial biological impact on the mechanisms of pathogenesis in cases of CNS compromise in CD patients coinfected by HIV [3–5].

In immunocompetent hosts, the apparent silent persistence of *T. cruzi* amastigote forms in the CNS has been challenged by the demonstration of behavioral alterations as sleep disorders, memory deficits, lower quality of life and depression, all of which have been reported in children and young adults during the chronic phase of CD [5,66,67]. Indeed, sleep disorders, memory deficit and depressive-like behavior were detected in chronically *T. cruzi*-infected animals [6,68]. Thus, it is tempting to propose that parasite products, cytokines (such as IFNγ and TNF) and *T. cruzi*-induced release of inflammatory mediators (like NO) may contribute to behavioral disturbs detected in chronically infected CD patients and experimental models of chagasic infection [5,6]. It has been demonstrated that inflammatory mediators as IFNγ, TNF, NO and others, mostly triggered by infectious agents (as *Mycobacterium* and *T. cruzi*) or theirs products, as LPS, are involved in behavioral abnormalities [6,69]. Furthermore, neuronal death might be the result of oxidative stress generated by the astrocyte dysfunction [70]. Consistently, oxidative damage is a common and early feature of Alzheimer’s disease, Parkinson’s disease, amyotrophic lateral sclerosis and other neurodegenerative disorders [25]. In these cases, the participation of proinflammatory cytokines have been demonstrated [71], alike amyotrophic lateral sclerosis, in which IFNγ contributes to the cross-talk between motoneurons and astrocytes, and has been proposed to contribute to motoneuron death by eliciting the activation of the lymphotoxin-β receptor (LT βR) through its ligand LIGHT [72,73]. In these states of injury and/or inflammation in the CNS, reactive astrogliosis (a series of molecular, functional and morphological events occurring in astrocytes) usually takes place [9,16], culminating among other responses in the production of NO. This molecule, in turn, leads to a state of oxidative stress, contributing to amplification and exacerbation of neurodegenerative processes [25]. Thus, we bring evidence that also in a parasitic disease as CD one should consider the glial disorders as intrinsic components of the neurodegenerative processes in an integrated view of pathological changes in the CNS [74], which may contribute to behavioral abnormalities.

## Conclusions

*In vivo* IFNγ expression was found in very close proximity with parasites in the CNS. Interestingly, *in vitro* IFNγ increased the susceptibility of astrocytes to *T. cruzi* infection and promoted amastigote growth. Altogether, these data suggest that in an inflammatory environment, composed of cytokines such as IFNγ, the entry and establishment of this parasite is facilitated, as may occur in the CNS, with the parasite being housed inside astrocytes. The infected astrocytes, in turn, affected by infectious and inflammatory damage, produce NO, which may contribute to sponsor neuronal damage/death. Therefore, our data suggest that IFNγ-dependent astrocyte susceptibility contributes to *T. cruzi* persistence in the CNS. Moreover, a Th1 immune response in this highly immunospecialized site or the perfusion of the CNS tissue by systemically produced IFNγ, in cases of blood-brain-barrier disruption, may fuel parasite growth and TNF and NO production, leading to a disturb in neuronal cell homeostasis and contributing to cell death.

## Supporting Information

S1 Fig Immunohistochemical staining of serial sections of the CNS showing close proximity of IFNγ^+^ cells and *T. cruzi* antigens with astrocytes and IFNγ^+^ cells in parallel to Iba1^+^ cells. (A) Serial section of the CNS of acutely (25 dpi) *T. cruzi*-infected mice showing astrocytes (left panel), parasite antigen^+^ areas (middle panel) and IFNγ^+^ cells (right panel). Sections of the CNS showing absence of staining when secondary antibodies are used but primary antibodies are omitted are also shown. (B) Serial sections of the CNS of acutely (25 dpi) *T. cruzi*-infected mice revealing absence of staining for CD3 (left panel), but proximity of Iba1^+^ cells (middle panel) and IFNγ-expressing cells (right panel). Representative pictures.
(TIF)

S2 Fig Establishment of primary astrocyte cell cultures of C3H/He mice. Representative pictures of (A) phase contrast and (B) Giemsa staining revealing adherent cells morphologically resembling astrocytes. (C). Immunohistochemical staining of primary cultures of CNS cells of C3H/He mouse showing presence of GFAP^+^ cells (revealed by anti-GFAP; green) and absence of CD11b^+^ cells (reveled by anti-CD11b^+^; red), supporting enrichment in astrocytes and absence of microglial cells. Nuclei are stained in blue with DAPI.
(TIF)

S3 Fig Pretreatment with IFNγ does not affect fibroblast infection by *T. cruzi*. (A) Experimental scheme showing that L-929 fibrobaslts were pretreated with IFNγ (10 ng/mL), subsequently infected using the MOI 1:1 or 10:1, and analyzed 4 or 24 hours post-infection (p.i.). The percentage of infected cells and the number of parasites per cells were analyzed at 4 hours (B and C) and 24 hours (D and E) at MOI 1:1 (B and D) or 10:1 (C and E).Data are presented as mean ± SEM of triplicates.
(TIF)

S4 Fig Effect of IFNγ increasing the growth of intracellular forms of *T. cruzi* in astrocytes is conserved in C57BL/6 mice. (A) Primary astrocyte cell cultures were infected with *T. cruzi* (MOI 1:1) for 4 hours. Afterwards, parasites were washed out, medium was replaced, and cells incubated for an additional 20 hours. Astrocytes bearing intracellular forms of *T. cruzi* were treated or not with IFNγ (10 ng/mL) and analyzed 24 hours later (48 hours post-infection, p.i.). (B) The graph shows the frequencies of *T. cruzi*-infected cells bearing different classes of parasite load. Data are presented as mean ± SEM of triplicates. *, *p* < 0.05 comparing not-treated (NT) with IFNγ-treated astrocytes.
(TIF)

S5 Fig Nitric oxide production by macrophages and astrocytes treated with IFNγ and/or LPS. (A) Experimental scheme showing that macrophages (RAW 264.7) or primary astrocyte cell cultures were treated or not with IFNγ (10 ng/mL) and/or LPS (10 ng/mL) and analyzed for NO production 24 and 48 hours after treatment. NO production by macrophages and primary astrocytes submitted to the different experimental conditions is shown in (B) and (C), respectively. (D) Effect of L-NAME on NO production inhibition in macrophages stimulated for 24 h with IFNγ and LPS. Data are presented as mean ± SEM of triplicates. *, *p* < 0.05, **, *p* < 0.01 and ***, *p* < 0.001 comparing IFNγ and/or LPS-treated macrophages and astrocytes with not-treated cells.
(TIF)
