# Supplementary figures and images for: Interferon-Gamma Promotes Infection of Astrocytes by Trypanosoma cruzi
Source: PLoS One. 2015 Feb 19;10(2):e0118600. doi: 10.1371/journal.pone.0118600 (PMC4335051; doi:10.1371/journal.pone.0118600)

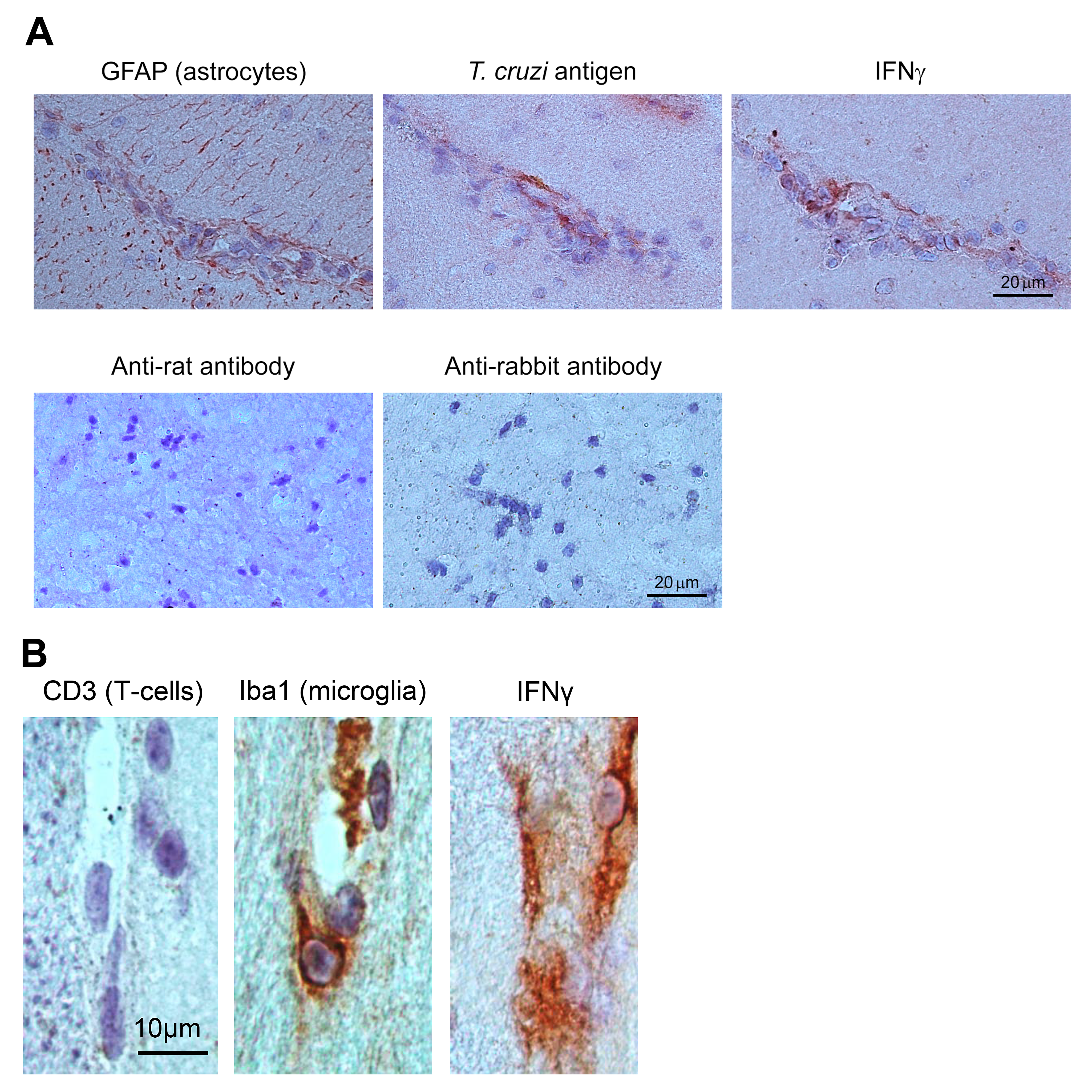

Supplement: S1 Fig — (A) Serial section of the CNS of acutely (25 dpi) T. cruzi-infected mice showing astrocytes (left panel), parasite antigen+ areas (middle panel) and IFNγ+ cells (right panel). Sections of the CNS showing absence of staining when secondary antibodies are used but primary antibodies are omitted are also shown. (B) Serial sections of the CNS of acutely (25 dpi) T. cruzi-infected mice revealing absence of staining for CD3 (left panel), but proximity of Iba1+ cells (middle panel) and IFNγ-expressing cells (right panel). Representative pictures. (TIF) [file pone.0118600.s001.tif]

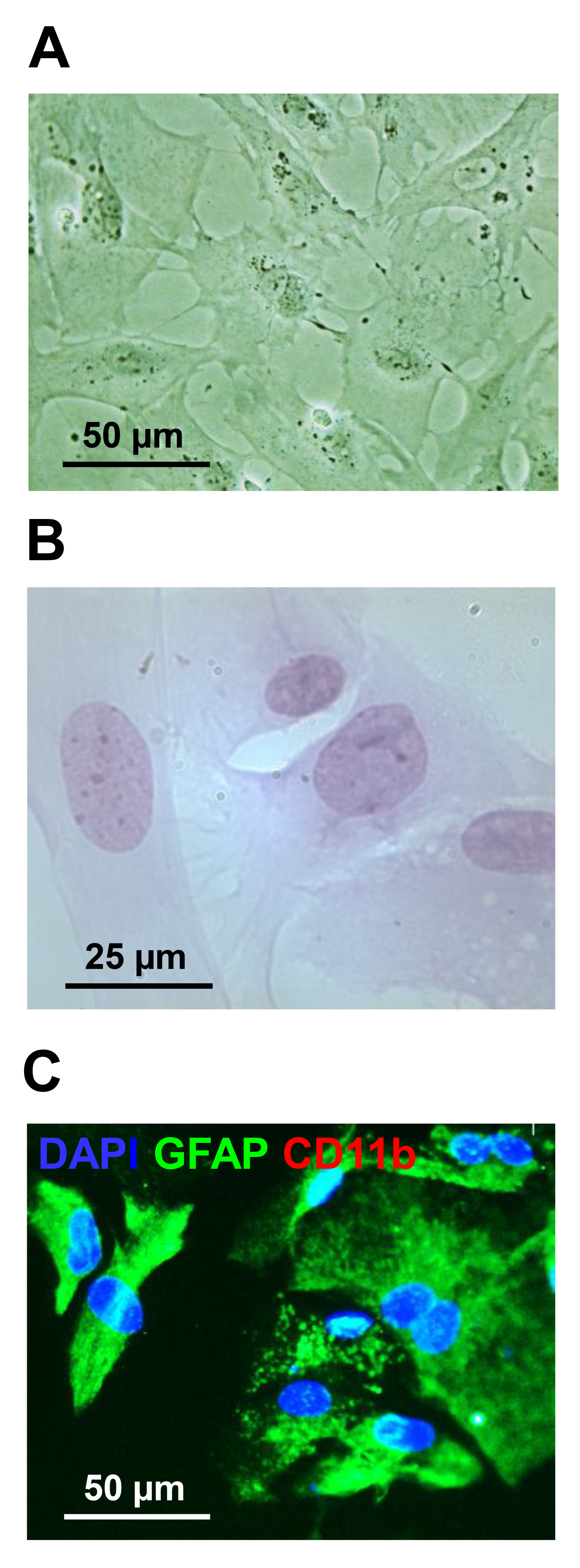

Supplement: S2 Fig — Representative pictures of (A) phase contrast and (B) Giemsa staining revealing adherent cells morphologically resembling astrocytes. (C). Immunohistochemical staining of primary cultures of CNS cells of C3H/He mouse showing presence of GFAP+ cells (revealed by anti-GFAP; green) and absence of CD11b+ cells (reveled by anti-CD11b+; red), supporting enrichment in astrocytes and absence of microglial cells. Nuclei are stained in blue with DAPI. (TIF) [file pone.0118600.s002.tif]

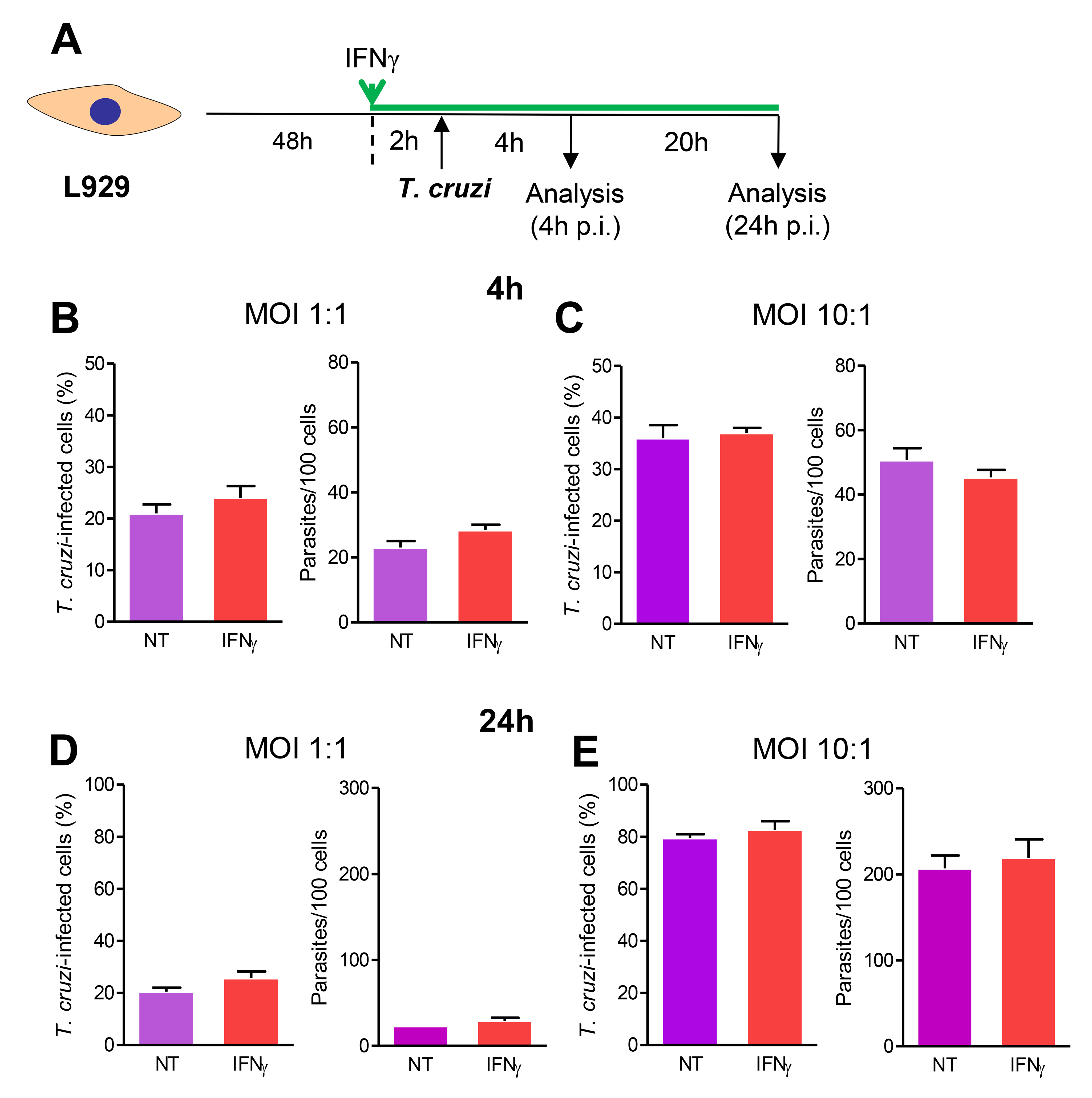

Supplement: S3 Fig — (A) Experimental scheme showing that L-929 fibrobaslts were pretreated with IFNγ (10 ng/mL), subsequently infected using the MOI 1:1 or 10:1, and analyzed 4 or 24 hours post-infection (p.i.). The percentage of infected cells and the number of parasites per cells were analyzed at 4 hours (B and C) and 24 hours (D and E) at MOI 1:1 (B and D) or 10:1 (C and E).Data are presented as mean ± SEM of triplicates. (TIF) [file pone.0118600.s003.tif]

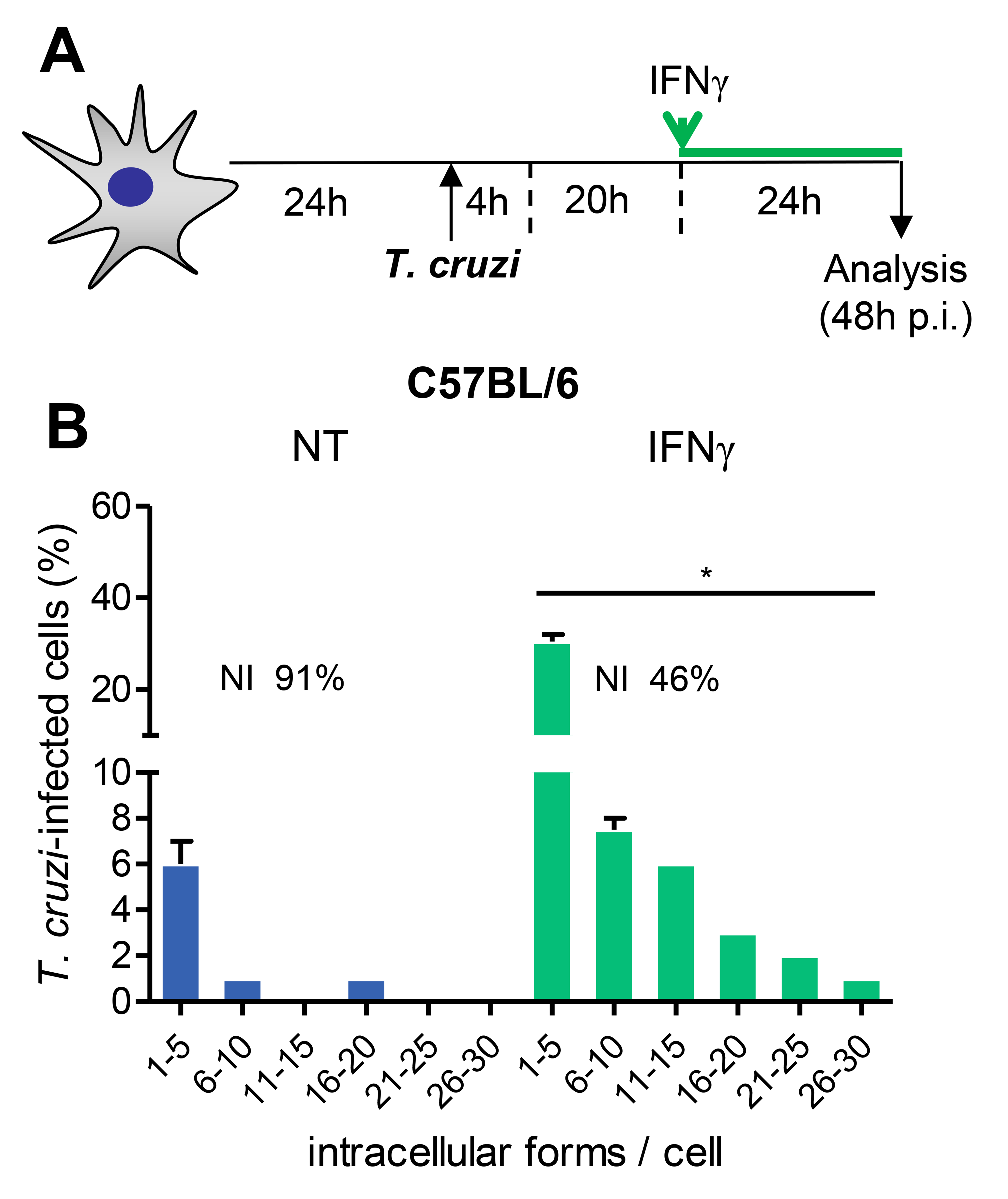

Supplement: S4 Fig — (A) Primary astrocyte cell cultures were infected with T. cruzi (MOI 1:1) for 4 hours. Afterwards, parasites were washed out, medium was replaced, and cells incubated for an additional 20 hours. Astrocytes bearing intracellular forms of T. cruzi were treated or not with IFNγ (10 ng/mL) and analyzed 24 hours later (48 hours post-infection, p.i.). (B) The graph shows the frequencies of T. cruzi-infected cells bearing different classes of parasite load. Data are presented as mean ± SEM of triplicates. *, p < 0.05 comparing not-treated (NT) with IFNγ-treated astrocytes. (TIF) [file pone.0118600.s004.tif]

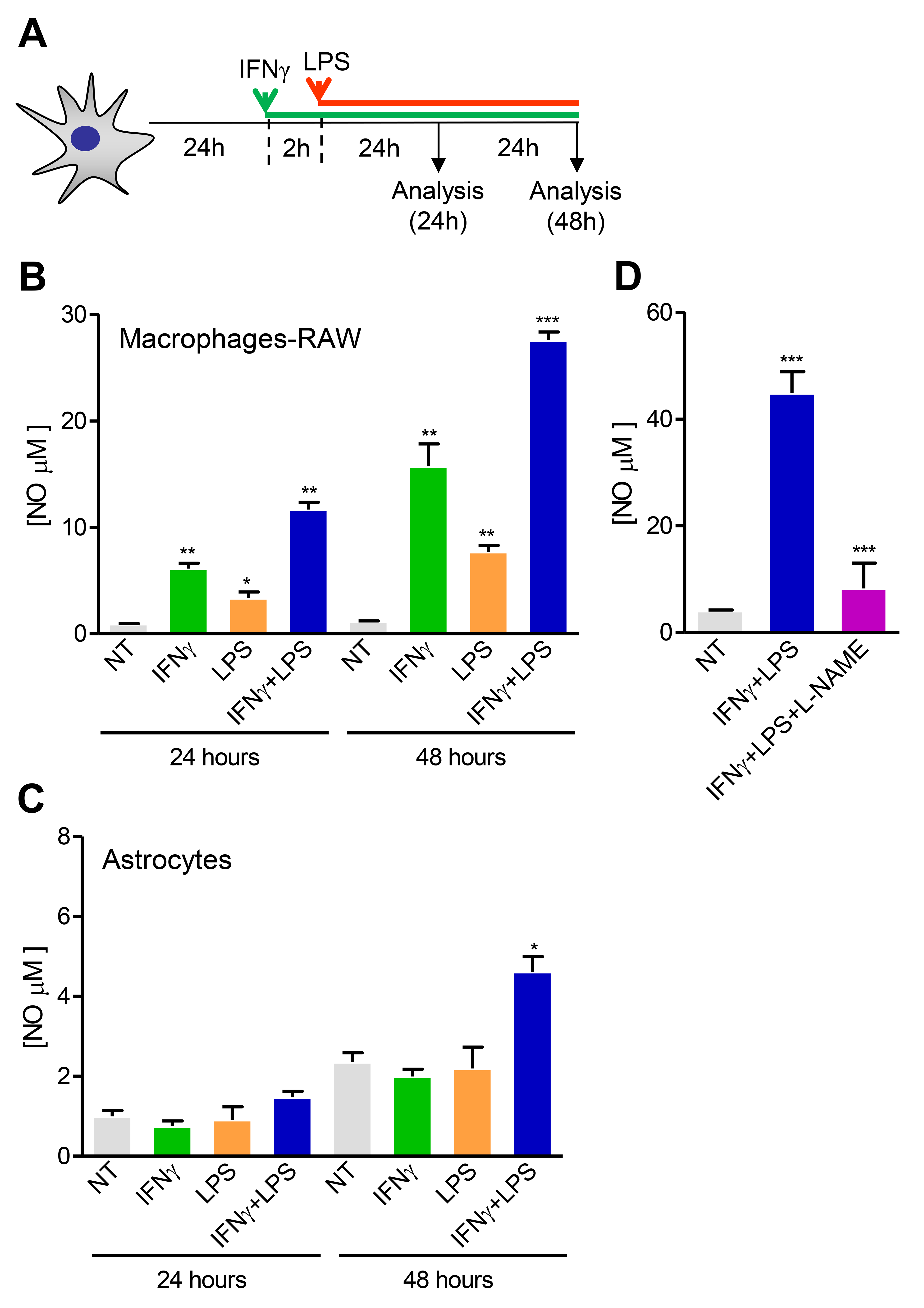

Supplement: S5 Fig — (A) Experimental scheme showing that macrophages (RAW 264.7) or primary astrocyte cell cultures were treated or not with IFNγ (10 ng/mL) and/or LPS (10 ng/mL) and analyzed for NO production 24 and 48 hours after treatment. NO production by macrophages and primary astrocytes submitted to the different experimental conditions is shown in (B) and (C), respectively. (D) Effect of L-NAME on NO production inhibition in macrophages stimulated for 24 h with IFNγ and LPS. Data are presented as mean ± SEM of triplicates. *, p < 0.05, **, p < 0.01 and ***, p < 0.001 comparing IFNγ and/or LPS-treated macrophages and astrocytes with not-treated cells. (TIF) [file pone.0118600.s005.tif]
